# Biomarkers of Muscle Metabolism in Peripheral Artery Disease: A Dynamic NIRS-Assisted Study to Detect Adaptations Following Revascularization and Exercise Training

**DOI:** 10.3390/diagnostics10050312

**Published:** 2020-05-16

**Authors:** Fabio Manfredini, Nicola Lamberti, Valentina Ficarra, Elpiniki Tsolaki, Sofia Straudi, Paolo Zamboni, Nino Basaglia, Vincenzo Gasbarro

**Affiliations:** 1 Section of Sports Sciences, Department of Biomedical and Surgical Specialties Sciences, University of Ferrara, Italy–Via Luigi Borsari 46, 44121 Ferrara, Italy; nicola.lamberti@unife.it; 2 Unit of Physical Medicine and Rehabilitation, Department of Neurosciences/Rehabilitation, University Hospital of Ferrara, Via Aldo Moro 8, 44124 Ferrara, Italy; sofia.straudi@gmail.com (S.S.); nino.basaglia@unife.it (N.B.); 3 Unit of Vascular and Endovascular Surgery, University Hospital of Ferrara, Via Aldo Moro 8, 44124 Ferrara, Italy; valentinaficarra@gmail.com (V.F.); niki.tsolaki@gmail.com (E.T.); vincenzo.gasbarro@unife.it (V.G.); 4 Vascular Diseases Center, University of Ferrara, Italy–Via Aldo Moro 8, 44124 Ferrara, Italy; zambo@unife.it

**Keywords:** peripheral artery disease, spectroscopy, near-infrared, exercise, rehabilitation, exercise testing

## Abstract

We assessed whether muscle metabolism biomarkers (MMb) identified by near-infrared spectroscopy (NIRS) are valid for determining adaptations following revascularization or exercise training in peripheral artery disease (PAD). Eighteen patients (males *n* = 13; 69 ± 7 years) were randomized to receive revascularization (Rev = 6) or pain-free home-based exercise (Ex = 12). MMb were safely collected via a NIRS-assisted treadmill test as area-under-curve for the spectra of oxygenated (-oxy), deoxygenated (-deoxy), differential (-diff) and total (-tot) hemoglobin traces. MMb, ankle–brachial index (ABI), pain-free (PFWD) and 6-min (6MWD) walking distances were assessed at baseline and after four months. MMb were correlated at baseline with ABI (MMb-oxy *r* = 0.46) and 6MWD (MMb-tot *r* = 0.51). After treatments, MMb-oxy showed an expected increase, which was more relevant for Rev group than the Ex (56% vs. 20%), with trends towards normalization for the other MMb. These changes were significantly correlated with variations in ABI (MMb-oxy *r* = 0.71; *p* = 0.002) and 6MWD (MMb-tot *r* = 0.58; *p* = 0.003). The MMb-diff in Rev group and MMb-deoxy in Ex group at baseline predicted clinical outcomes being correlated with PFWD improvements after 4-month (*r* = −0.94; *p* = 0.005 and *r* = −0.57; *p* = 0.05, respectively). A noninvasive NIRS-based test, feasible in a clinical setting, identified muscle metabolism biomarkers in PAD. The novel MMb were associated with validated outcome measures, selectively modified after different interventions and able to predict long-term functional improvements after surgery or exercise training.

## 1. Introduction

Peripheral artery disease (PAD) affects millions of people worldwide, particularly elderly individuals [1,2]. In a high percentage of cases, patients suffer from reduced mobility due to the presence of limiting symptoms such as claudication or exertional pain [1]. At this stage, medical therapy, exercise and endovascular therapy or surgery [2,3] are options to improve walking ability and quality of life as well reduce the risk of cardiovascular events and vascular worsening [2,4].

Hemodynamic severity and functional capacity are generally assessed in clinical practice by the ankle–brachial index (ABI) and treadmill or ground walking tests, which estimate patients’ peripheral blood supply and walking capability, respectively [3,5]. These validated measures are also used to evaluate hemodynamic and functional outcomes after rehabilitation or revascularization [4]. However, these methods, being a static hemodynamic measurement and a global measure of functioning, respectively, may poorly reflect the peripheral changes that occurred. In addition to blood pressure changes, other local hemodynamics (e.g., collateralization, vascular reactivity and microcirculation) may influence muscle perfusion [6,7,8,9]. Different techniques may determine the perfusion patterns in PAD [4], including conventional imaging techniques routinely used in clinical practice in the diagnostic or preoperative phase, such as digital subtraction angiography [3,5]. In addition new methods specifically proposed may detect changes in muscle perfusion muscles after revascularization of the lower limbs, such as positron emission tomography, magnetic resonance imaging or the ASL technique [3,10,11,12,13].

However, these methods, which are progressively more attractive, are limited in their potential routine use by the frequent need to administer radioactive or contrast products [13], by the availability of sophisticated instruments and skilled personnel and by high costs. Finally, these measures are static or with a dynamic phase limited to perfusion hyperemia [3].

Moreover, in addition to perfusion, other peripheral factors (i.e., mitochondrial function, type of fibers, etc.) contribute to muscle metabolism by improving or affecting oxygen utilization with a direct impact on functional outcomes [7,14].

Considering all these aspects, support in the study of peripheral adaptations is offered by the use of near-infrared spectroscopy (NIRS). This technique noninvasively explores tissue microvascular hemodynamics, monitoring the local balance between oxygen delivery and consumption [15,16,17,18,19]. The NIRS technique, though it does not allow exploration of the perfusion of deep tissues and monitors only a limited muscle region, offers a rapid, noninvasive dynamic study of the changes in perfusion, skeletal muscle blood flow and mitochondrial respiratory function [20].

Since NIRS represent a useful technique for both dynamic and bedside measurements [17,19], several authors have previously employed it in PAD patients, studying in vivo skeletal muscle oxidative function [15,19,21]. The technique has also been applied to monitor oxygenation during treadmill test [22,23], in response to endovascular revascularization [24] or home-based walking programs [25] Finally, NIRS was used to determine the perfusion of several muscular district in response to motor tasks [7] or use of devices as intermittent pneumatic compression or elastic stockings [26,27,28,29]. However, to the best of our knowledge, no study has compared metabolic adaptations occurring after revascularization or exercise in the walking muscles of patients with claudication.

After validating the quantification of muscle perfusion changes by a NIRS-assisted treadmill test [22], we used this method to study the different adaptations occurring following walking programs [25] as well to highlight the different symptoms perception in persons with diabetes [30]. This study aims to test the hypothesis that different muscular adaptations may be quantified and selectively recognized after revascularization or home-based exercise in PAD patients.

The present study represents a secondary analysis of a pilot randomized trial in PAD patients with intermittent claudication [29], including active subjects following successful revascularization and patients performing a pain-free structured home-based walking program. The aim of the study was to determine whether muscle metabolism biomarkers (MMb) identified by a NIRS-based treadmill test [22] performed in a clinical setting are potentially suitable to quantify muscle adaptations in PAD as they are (i) correlated with validated measures; (ii) selectively modified by different interventions; and (iii) able to predict functional improvements following revascularization or exercise training.

## 2. Materials and Methods

To assess the metabolic muscle adaptations induced by different interventions in PAD, we studied a group of patients randomized in a previous single-center clinical pilot trial [29] that took place at the Departments of Vascular Surgery and Rehabilitation Medicine of the University Hospital of Ferrara. The study was approved by the local ethics committee (code: AOUFe/Sper/137/11, approved on July 2011), and written informed consent was obtained from all participants.

### 2.1. Participants

Eligible participants (*n* = 18; males *n* = 13; 69 ± 7 years) were identified from a cohort of consecutive new patients with PAD and intermittent claudication. The inclusion criteria were as follows: males and females aged 60–80; Rutherford category 2 or 3; initial claudication distance <100 m; one or more arterial stenoses at aorto–iliac or femur–popliteal district with >50% reduction in diameter requiring revascularization. The exclusion criteria were as follows: abdominal aortic aneurysm; clinical conditions limiting treadmill testing; incapacitating cardiac disease or any other condition contraindicating revascularization procedures; anemia and hemoglobinopathies; and other disorders masking or confounding vascular symptoms (e.g., neurological claudication) [29].

### 2.2. Interventions

Patients were randomized with a 2:1 ratio to receive structured home-based exercise training (Ex) or peripheral revascularization (Rev).

The noninvasive intervention was based on the structured home-based walking test in–train out program prescribed at the hospital and performed at home [31,32]. The program included two daily 10-min sessions of intermittent walking (1:1 walk:rest ratio) at a slow speed, performed at home paced by a metronome. The walking speed of each exercise bout was increased weekly.

Revascularization procedures were performed at the Department of Vascular Surgery. For each patient, the option that was most likely to yield the best hemodynamic improvement (i.e., endovascular, open or hybrid) was selected by the surgeon. After the intervention, the patients received standardized recommendations to be physically active at home according to the guidelines and they underwent a clinical and instrumental evaluation.

### 2.3. Outcome Measures

Outcome measures were assessed at baseline (T0) and 4 months (T4) from the day of the intervention or the beginning of the Ex program. We collected baseline demographic data. All of the measurements were taken in the afternoon between 13:30 and 16:30 in a temperature-controlled environment and separated by 10-min intervals. The same expert outcome assessor, not blinded to the treatments, performed all the measurement sessions.

#### 2.3.1. Hemodynamics

The ankle–brachial index was measured according to published standards [33]. The leg with the lowest ABI value was considered for the present study. The vessels were considered “not compressible” for ABI measurements >1.40 or cuff pressure values >300 mmHg with a Doppler signal still present.

#### 2.3.2. Physical Functioning

The 6-min walking test was performed to determine patients’ pain-free walking distance (PFWD) and 6-min walking distance (6MWD) [34]. Patients were instructed to walk back and forth along a 22 m corridor with the aim of covering as much ground as possible in 6 min.

#### 2.3.3. Muscle Metabolism and Related Biomarker Assessment

An incremental treadmill test [22,35] supported by near-infrared spectroscopy (NIRS) was performed.

A continuous wave NIRS system (Oxymon MK III Artinis Medical System, The Netherlands), consisting of two channels using intensity-modulated light at 1 Hz frequency and 3 wavelengths (905, 850 and 770 nm) corresponding to high absorption of oxyhemoglobin (oxy) and deoxyhemoglobin (deoxy), was employed. Near-infrared light propagating through biologic tissue is partly absorbed or scattered by the tissues and partly recollected by the detector; therefore, the intensity of the recollected light provides information about oxy- and deoxyhemoglobin concentrations. From these changes, additional parameters were calculated as the sum of oxy- and deoxy- or total hemoglobin (tot) and the difference between oxy- and deoxy- or differential hemoglobin (diff). After taking skinfold measurements, NIRS optodes with an interoptode distance of 3.5 cm, allowing light penetration of approximately 20 mm, were placed on the medial side of the calf (gastrocnemius muscles) and secured with tape.

Patients were asked to perform a treadmill test [22,35] based on level walking with a slow initial speed of 1.5 km/h and increments of 0.1 km/h every 10 m until reaching the maximal speed attainable (Smax), as limited by symptoms or fatigue.

**Figure 1:**
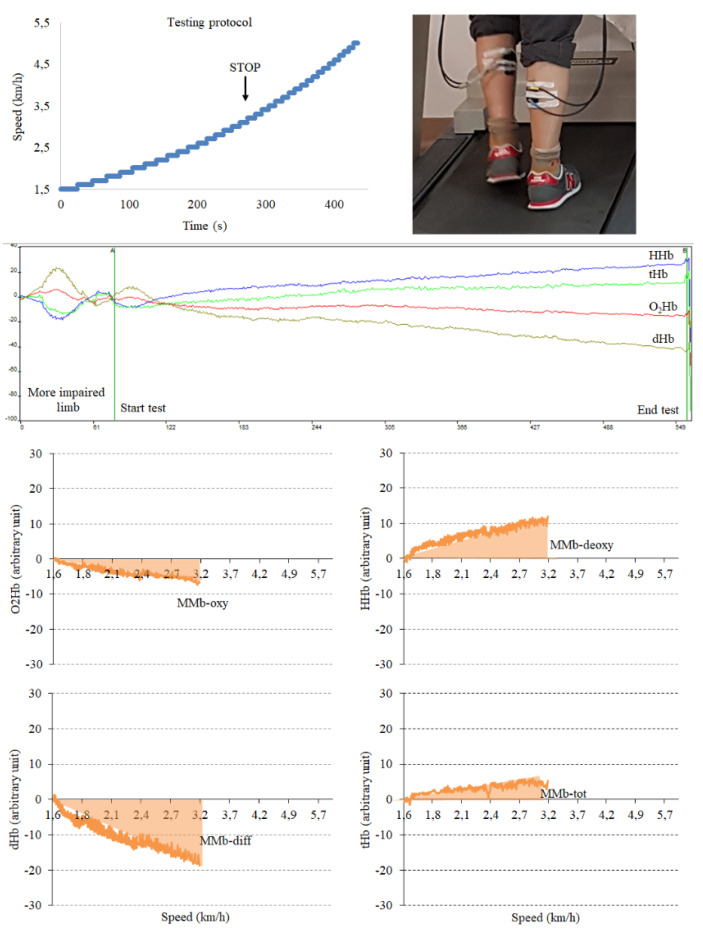
Process of testing, acquisition, elaboration and calculation of the area-under-curve (muscle metabolism biomarkers, MMb) for the four hemoglobin traces.

At the end of the testing session, semiquantitative data collected by the NIRS instrument were analyzed using the software Oxysoft 2.0.47 (Artinis Medical System, The Netherlands). The traces obtained are shown in Figure 1.

To quantify the variations, after assigning the first value (beginning of the test) of each hemoglobin trace test to be 0, the area under the curve (AUC) for each variable was calculated by summing all the single values obtained until the end of the test. The parameters considered were the AUC of oxy-, deoxy-, diff- and tot–hemoglobin that were calculated for the more diseased limb of each patient, as determined by the lower ABI value or the worst duplex ultrasound examination record in case of incompressible vessels. For this study, the AUCs were considered muscle metabolism biomarkers (MMb) for each of the four hemoglobin traces: MMb-oxy, MMb-deoxy, MMb-diff and MMb-tot.

At T4, when the treadmill test were longer with respect to baseline, the AUC calculation was stopped at the exact second when the test at T0 ended to ensure the same length for each hemoglobin trace.

### 2.4. Statistical Analysis

The data distribution was verified by the Shapiro–Wilk test. Continuous variables are expressed as the mean ± standard deviation or median (interquartile range), and categorical variables are expressed as numbers and percentages.

The characteristics of the study groups were compared using unpaired Student’s *t*-tests, Mann–Whitney tests or chi-squared tests, as appropriate. Differences between groups were assessed by Mann–Whitney test or unpaired Student’s *t*-tests, as appropriate. Within-group variations were verified by paired-sample *t*-tests or Wilcoxon tests.

Correlations between study variables were assessed by means of Spearman’s rho. A *p* value ≤ 0.05 was considered statistically significant. Data were analyzed using MedCalc Statistical Software version 19.1.5 (MedCalc Software., Ltd., Ostend, Belgium). 

Data availability: Research data are available at: http://dx.doi.org/10.17632/r3sxt3m8xj.1.

## 3. Results

Of the 27 patients enrolled in the study, 18 participants completed the scheduled interventions and the 4-month testing session. Twelve patients belonged to the Ex group and six to the Rev group. A flow diagram of the participants is reported elsewhere [29]. The two groups did not differ at baseline in terms of anthropometrics, comorbidities, hemodynamics, performance or muscle metabolism parameters assessed by NIRS (Table 1).

### 3.1. Interventions

All patients randomized to the Ex group completed the rehabilitation treatment without any adverse events. As reported in the logbooks, patients completed 218 ± 49 exercise sessions, for a total of approximately 34 h of walking exercise over 4 months. Patients in the Rev group underwent revascularization procedures (2 endovascular (angioplasty), 2 surgical (bypass in autologous vein), 2 hybrid (thromboendarterectomy plus angioplasty)) without adverse outcomes. No changes in therapy were reported, except for the dual antiplatelet therapy prescribed. At T4, they verbally reported being active after the interventions, referring to having performed a bout of at least 20 min of exercise at a self-selected speed for a median of 2 days per week.

### 3.2. Hemodynamics and Functional Capacity

Baseline data are reported in Table 1. The baseline ABI was not correlated with functional parameters (*r* = 0.23 *p* = 0.40 for PFWD and *r* = 0.41 *p* = 0.13 for 6MWD).

After the interventions, the ABI significantly increased in both groups, with a significantly between-group difference in favor of the Rev group. Similarly, the PFWD and 6MWD increased in both groups, with a significant difference between groups in favor of the Rev group for the 6MWD only. The data are summarized in Table 2.

### 3.3. Muscle Metabolism Biomarkers

The skin folds for the calf were less than 10 mm for all participants, allowing for reliable measurement of the region by the NIRS system. All patients completed the testing sessions without local or general adverse effects during the NIRS measurements.

The NIRS parameters exhibited variations after both interventions. In the Rev group, the MMb represented by the AUC of the hemoglobin traces collected with the NIRS system showed a trend towards normalization (Figure S1).

**Figure 2:**
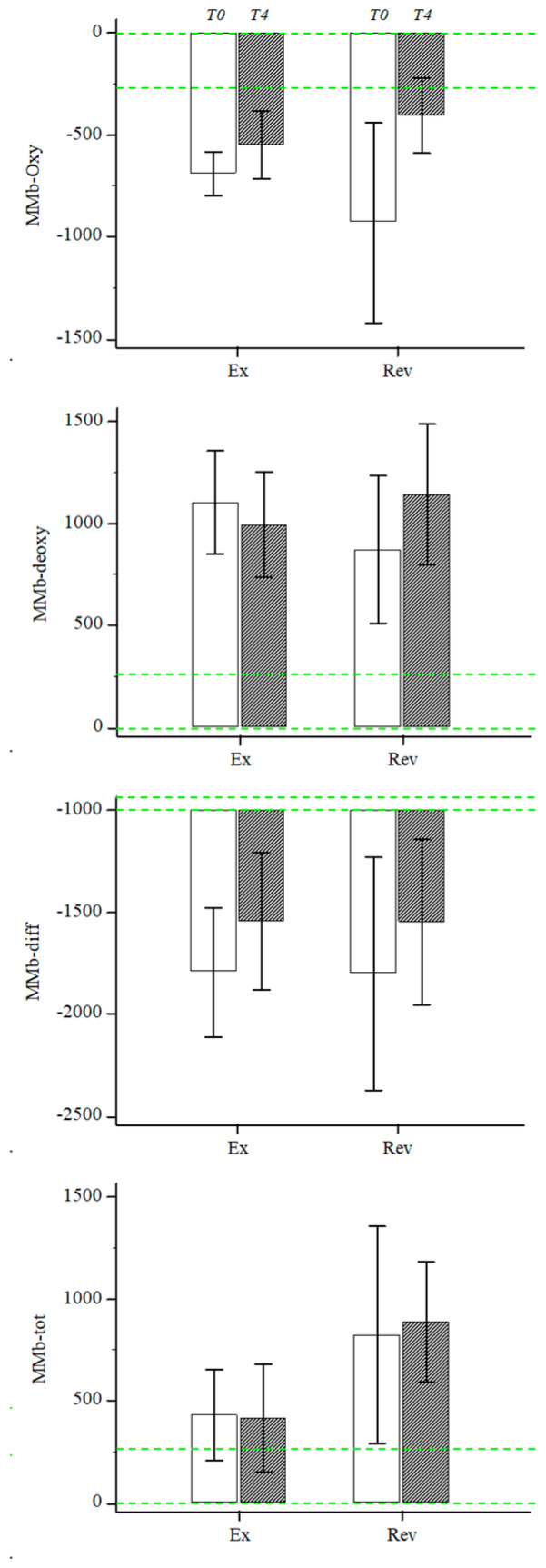
Muscle metabolic biomarkers for the two groups obtained at baseline (white column) and after 4 months (gray column). Green line represent the reference values in a healthy population previously tested [25]. Data are expressed as mean and standard error of mean. Rev: revascularization; Ex: exercise; T0: baseline; T4: 4 months.

A marked reduction in MMb-oxy (56%) and changes in the other AUCs of 12%, 31% and 7% for MMb-diff, MMb-deoxy and MMb-tot, respectively, were observed, even in the absence of statistically significant variations (Figure 2).

A similar trend towards normalization was observed in the Ex group for all four biomarkers without significant variations. The changes observed were 20%, 19% 10% and 2% for MMb-oxy, MMb-diff, MMb-deoxy and MMb-tot, respectively (Figure 2).

Despite no significant between-group differences, different magnitudes of changes were observed for MMb. In particular, a 3-fold variation was observed for MMb-oxy in the Rev group compared to the Ex group; conversely, a two-fold variation in MMb-deoxy was observed in the Ex group vs. the Rev group.

### 3.4. Relationship between Validated Outcome Measures and MMb

At baseline, in the whole population, a correlation between NIRS biomarkers and validated outcome measures was observed, particularly between MMb-oxy (*r* = 0.46; *p* = 0.071) and ABI, MMb-oxy and PFWD (*r* = −0.41; *p* = 0.041) and MMb-tot and 6MWD (*r* = 0.51; *p* = 0.003).

In the whole population, after the interventions the changes in some NIRS parameters were significantly correlated with those observed in the validated parameters.

**Figure 3:**
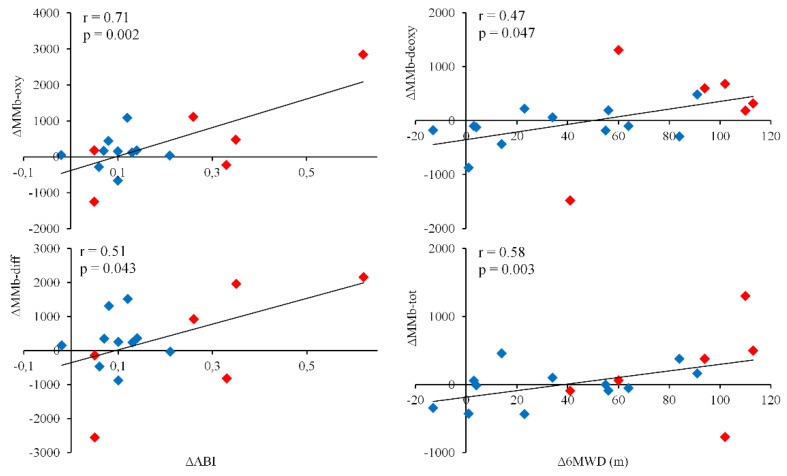
Rank correlation between variations of MMbs and variations of hemodynamic and functional parameters after interventions. Red: Rev group; Blue: Ex group; ABI: ankle–brachial index; 6MWD: 6-min walking distance.

Specifically, variations in MMb-oxy and MMb-diff were significantly correlated with changes in ABI. Moreover, variations in MMb-deoxy and MMb-tot were directly correlated with improvements in 6MWD (Figure 3), but not ABI changes (*r* = 0.11; *p* = 0.69).

When analyzing the response to different interventions, despite the reduced sample size, the changes in ABI were correlated with MMb-diff (*r* = 0.78; *p* = 0.065) variations in the Rev group and with MMb-tot (*r* = 0.60; *p* = 0.043) variations in the Ex group.

### 3.5. Relationship between Baseline Values of MMb and Changes in Functional Capacity after the Interventions

**Figure 4:**
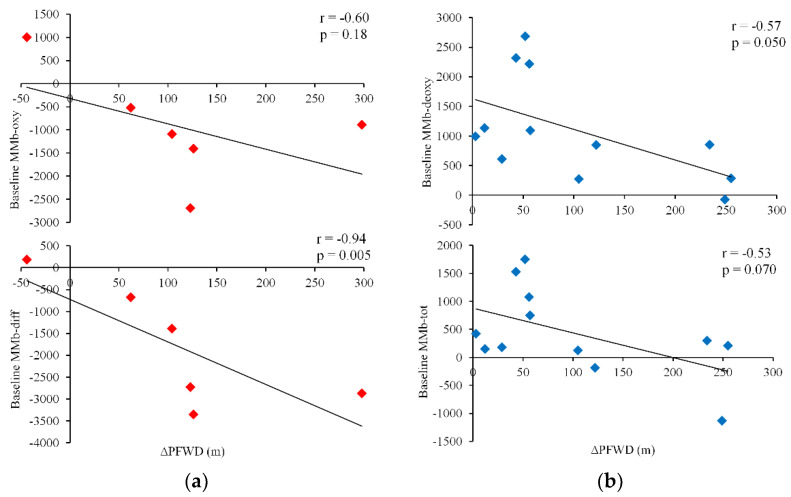
Predictive correlations between baseline values of MMb and variations of pain-free walking distance 4-month after interventions. (**a**) revascularization; (**b**) exercise. PFWD: pain-free walking distance.

In the Rev group, the baseline values of MMb-diff were strongly correlated with variations in PFWD observed after revascularization (*r* = −0.94; *p* = 0.005). A moderate statistically insignificant correlation was noted for MMb-oxy (*r* = −0.60; *p* = 0.18) (Figure 4a).

In the Ex group, baseline MMb-deoxy and MMb-tot were correlated with improvements in PFWD (*r* = −0.57, *p* = 0.05; and *r* = −0.53, *p* = 0.07, respectively) (Figure 4b).

Conversely, no significant correlations were observed between traditional outcome measures (ABI, PFWD and 6MWD) at baseline and variations in PFWD and 6MWD after the interventions in the whole population or in either groups.

## 4. Discussion

This study identified novel muscle metabolism biomarkers that allowed to quantify and compare peripheral muscle adaptations occurring in active patients with PAD four months after a revascularization intervention or the start of a home-based pain-free walking program. The NIRS biomarkers correlated with validated parameters and predicted the recovery of mobility following two interventions in a group of patients.

A testing method that was previously described [22] has been successfully utilized to determine, without any limitation in terms of tolerability, execution and analysis, the value of the AUC of the different parameters obtained with an NIRS device. Two of these parameters are directly determined (i.e., oxy- and deoxyhemoglobin, which represent the amount of hemoglobin with or without oxygen, respectively) and thus represent the local degree of oxygenation of the region of muscle under the sensors. Two additional parameters are calculated (i.e., diff- and tot–hemoglobin) and describe the local degree of arteriovenous difference and of blood volume [18,19]. These four biomarkers, obtained during an incremental treadmill test, allowed us to describe the peripheral impact of the interventions in the two groups. Testing methods assisted by NIRS were previously proposed, but they were mainly used to assess the presence of diseases [14,15,16] rather than to study the effects of specific interventions.

However, a previous study based on the same NIRS-assisted test as that employed in the present study distinguished between local exercise-induced adaptations occurring in two groups of PAD patients and in healthy subjects [25]. The study also highlighted selective and favorable NIRS adaptations and hemodynamic changes mostly in patients following a structured pain-free walking program similar to the program proposed here, rather than in patients advised to walk at a spontaneous speed in the presence of pain [25].

In the present study, the MMb showed improved dynamic oxygenation at the gastrocnemius following both the invasive and noninvasive interventions, as evidenced by a reduction in the values of MMb-oxy in both groups after four months. In particular, an expected higher oxygen supply in the Rev group than in the Ex group was observed, as witnessed by the oxygenation area, which improved 2-fold (56% compared to 20%) without statistical significance due to the small sample size and the large standard deviations determined.

Interestingly, the variations in MMb-oxy were congruent and highly correlated with the variations in ABI observed in the more impaired limb in the whole population and separately in the two groups despite the small sample size.

These variations in ABI and muscle perfusion, as expected, were largely superior in the Rev group, being obtained with different revascularization procedures and in some cases related to multiple districts.

However, an increase in ABI was also present and measurable in the Ex group, with an average variation of approximately 15%. The ABI increase, not reported after supervised exercise programs [4], but previously reported by our research group [25,29,32] and found to be clinically relevant in the long term [36,37], is here confirmed by the increase in muscle oxygenation. Such improvements may derive from a favorable stimulus (in terms of intensity/volume) evoked by the walking program and mediated by shear stress on endothelial cells with endothelial nitric oxide synthase phosphorylation [38].

In addition, in the Ex group, the NIRS technique also showed better aerobic efficiency in terms of oxygen utilization, with a larger improvement in the median differential hemoglobin area than the Rev group (19% vs. 12%, respectively). Adaptations in mitochondrial function accounting for the functional improvements observed were recently reported following an exercise training assisted by NIRS at moderate deoxygenation [39]. Interestingly, this type of training has evident affinities with the training program prescribed to the Ex patients [31,32,40]. In this group, according to the information derived from biomarkers, the significant recovery of mobility is the result of relatively modest, but significant hemodynamic improvement combined with greater efficiency of utilization of the oxygen itself, even after only four months of the program.

A further interesting observation is represented by the fact that MMb expressed selective adaptations in response to different interventions. Moreover, their changes were also correlated with walking ability at baseline, unlike the ABI. As a further point of interest, the changes in NIRS markers were supported in their validity by their correlation with the ABI and performance variations.

In this respect, a further intriguing aspect is represented by the predictive ability of the NIRS values determined at baseline for the measured individual functional recovery. In the Rev group, only the NIRS biomarkers, not the ABI and functional baseline values, were correlated with the changes in walking performance, especially the PFWD at four months, suggesting the possibility to estimate performance changes after revascularization and therefore define the benefit–cost ratio for patients.

The study has several limitations. The main weakness is the sample size and the different sample size in each group, derived from randomization. In addition, the limitations related to the NIRS technique must be considered. We analyzed only a localized region of a particular muscle, but oxygen saturation may vary within and between specific muscles [41]. In general, local skinfolds or edema may also affect the optical reading. Furthermore, even if affixed by the same expert operator, the sensors may be positioned at the same point (measurements and photos) for the same subject, but at slightly different locations for different subjects. This study measured long-term adaptations in the Rev group so that they could be compared with results from an acceptable duration of the walking program in the Ex group. Furthermore, NIRS measurements immediately after surgery could be less reliable due to the presence of surgical wounds or postoperative edema. Finally, the walking activity performed by the patients in the Rev group was not documented and was based only on their oral report.

From a practical point of view, a non-invasive NIRS-assisted test allowed to measure and compare at the same time both muscle oxygen availability and utilization following different interventions. In addition, the test may estimate the medium-term benefits on mobility before revascularization, thus enabling the vascular surgeon and the patient with claudication to evaluate the risk-benefit ratio.

## 5. Conclusions

The study confirms that the NIRS-based assessment of perfusion at the calf muscle following interventions may provide additional information than that obtained by the ABI and walking parameters. An incremental test assisted with the NIRS technique, performed in a rehabilitative setting quickly and safely produced different biomarkers. These MMb showed a selective response to each intervention, with variations that were congruent and associated with validated hemodynamic and functional parameters. In addition, only these novel biomarkers showed a correlation with functional outcomes four months after surgery and exercise training. The present novel results need to be confirmed in a larger study.

## Figures and Tables

**Table 1:** Baseline characteristics of patients in the two study groups.

Variable		Rev (*n* = 6)	Ex (*n* = 12)	*p* Value
Age, years		69 ± 8	68 ± 5	0.78
Male sex, n (%)		6 (100)	7 (58)	0.07
Body mass index, kg/m^2^		25 ± 5	27 ± 3	0.27
PAD duration, years		8 ± 6	8 ± 7	0.97
Risk factors, n (%)				
	Hypertension	6 (100)	11 (92)	0.48
	Hyperlipidemia	4 (67)	7 (58)	0.74
	Diabetes	2 (33)	5 (42)	0.74
	Smoking	6 (100)	11 (92)	0.48
	Family history	2 (33)	3 (25)	0.72
Comorbidities, n (%)				
	Myocardial infarction	3 (50)	2 (18)	0.15
	Coronary heart disease	4 (67)	4 (33)	0.19
	Cerebrovascular disease	0 (0)	0 (0)	-
	Lung disease	0 (0)	1 (8)	0.48
	Lower limb revascularizations	2 (40)	4 (33)	0.80
	Charlson comorbidity index	6 ± 2	6 ± 2	0.82
Therapy, n (%)				
	Anticoagulants	1 (20)	2 (16)	0.87
	Antiplatelets	6 (100)	11 (92)	0.48
	Cilostazol/pentoxifylline	0 (0)	0 (0)	-
	Antihypertensive	6 (100)	11 (92)	0.48
	Statins	4 (67)	6 (50)	0.27
	Hypoglycemic agents/insulin	2 (33)	5 (42)	0.74
Lesion location, n (%)				
	Aortoiliac	4 (67)	4 (33)	0.48
	Femoropopliteal	6 (100)	12 (100)	-
	Infrapopliteal	2 (33)	5 (42)	0.87
Outcome measures				
	ABI, more impaired limb	0.57 ± 0.17	0.56 ± 0.19	0.99
	ABI, less impaired limb	0.85 ± 0.27	0.81 ± 0.19	0.76
	PFWD, m	137 ± 41	173 ± 126	0.51
	6MWD, m	306 ± 102	312 ± 67	0.88
	Maximal speed, km/h	3.1 ± 0.9	3.2 ± 1.0	0.83
NIRS parameters				
	MMb-oxy	−930 ± 1204	−691 ± 372	0.83
	MMb-deoxy	874 ± 886	1104 ± 872	0.39
	MMb-diff	−1804 ± 1398	−1795 ± 1096	0.83
	MMb-tot	826 ± 1304	432 ± 774	0.61

Rev: revascularization; Ex: exercise; PAD: peripheral artery disease; ABI: ankle–brachial index; PFWD: pain-free walking distance; 6MWD: 6-min walking distance: HR: heart rate: MMb muscle metabolism biomarker; -Oxy: oxygenated hemoglobin; -deoxy: deoxygenated hemoglobin; -diff: differential hemoglobin; -tot: total hemoglobin.

**Table 2:** Outcome measures values before and after interventions.

Variable	Rev (*n* = 12)			Ex (*n* = 6)			Between Groups	
	T0	T4	∆	T0	T4	∆	∆	*p* Value
ABI	0.57 (0.38–0.75)	0.84 * (0.70–0.99)	0.28 (0.05–0.50)	0.56 (0.43–0.70)	0.66 * (0.53–0.80)	0.10 (0.06–0.14)	0.18 (0.10–0.26)	0.025
PFWD, m	137 (94–179)	248 * (141–355)	111 (−5 +228)	173 (92–253)	275 * (187–362)	101 (42–160)	10 (−95 +115)	0.84
6MWD, m	306 (198–413)	392 * (285–499)	87 (56–118)	312 (269–354)	346 * (292–400)	35 (13–57)	52 (17–87)	0.006
MMb-oxy	−930 (−2117 −89)	−406 (−875 +64)	525 (−924 +1974)	−691 (−927 −454)	−550 (−915 −184)	141 (−154 +435)	384 (−530 +1298)	0.39
MMb-deoxy	874 (−56 +1803)	1143 (261 −2025)	270 (−718 +1257)	1104 (550–1658)	994 (430–1557)	−110 (−329 +109)	379 (−255 +1014)	0.22
MMb-diff	−1804 (−3271 −336)	−1549 (−2594 −503)	255 (−1632 +2142)	−1795 (−2491 −1098)	−1544 (−2280 −807)	250 (−232 +733)	5 (−1251 +1262)	0.99
MMb-tot	826 (−543 +2194)	889 (131 −1646)	63 (−1008 +1134)	432 (−60 +924)	415 (−166 +996)	−17 (−198 +164)	80 (−574 +374)	0.80

Rev: revascularization; Ex: exercise; ∆: difference; ABI: ankle–brachial index; PFWD: pain-free walking distance; 6MWD: 6-min walking distance; T0: baseline; T4: 4-month; MMb muscle metabolism biomarker; -Oxy: oxygenated hemoglobin; -deoxy: deoxygenated hemoglobin; -diff: differential hemoglobin; -tot: total hemoglobin. Data are expressed as mean (95% confidence interval). * Paired samples *t*-test or Wilcoxon test *p* < 0.05.

## Supplementary Materials

The following are available online at https://www.mdpi.com/2075-4418/10/5/312/s1, Figure S1: Representation of oxygenated hemoglobin trace and MMb-oxy (colored area) at baseline (a) and after interventions (b) in one patient for each group.
