# Supplementary material for: Biomarkers of Muscle Metabolism in Peripheral Artery Disease: A Dynamic NIRS-Assisted Study to Detect Adaptations Following Revascularization and Exercise Training
Source: Diagnostics (Basel). 2020 May 16;10(5):312. doi: 10.3390/diagnostics10050312 (PMC7277989; doi:10.3390/diagnostics10050312)
Supplement: Supplementary file 1 [file diagnostics-10-00312-s001.pdf]

Supplementary Material

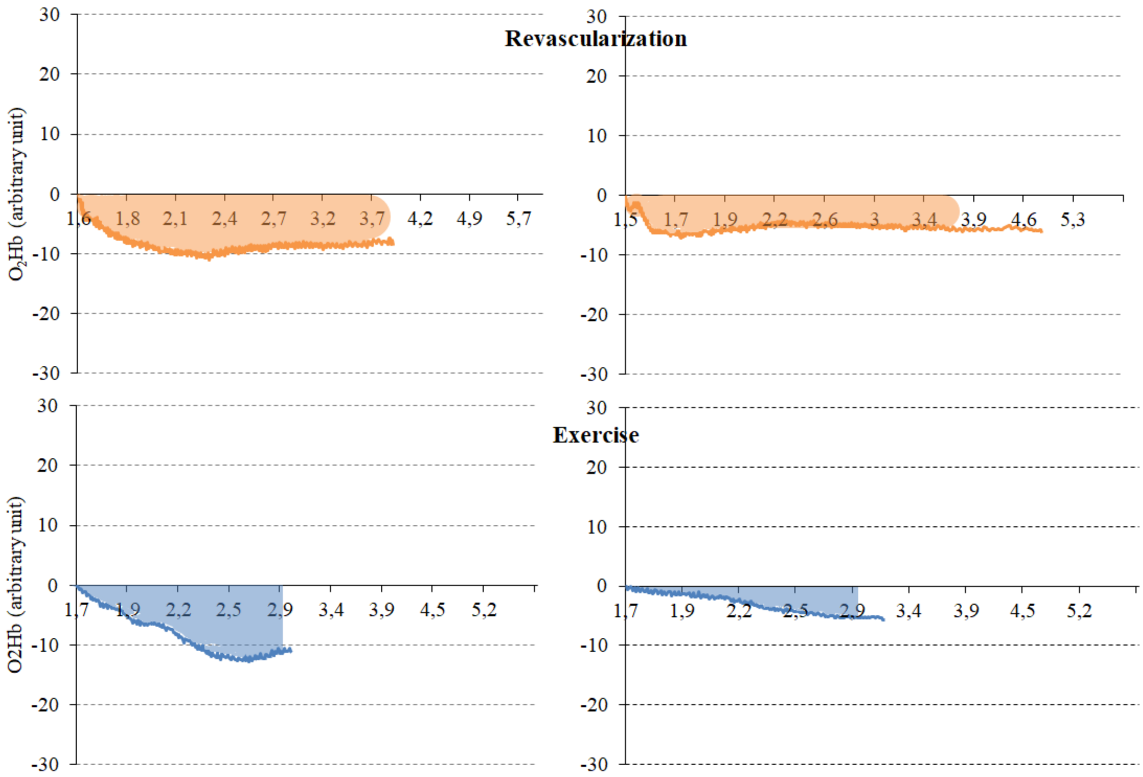

Figure S1. Representation of oxygenated hemoglobin trace and MMb-oxy (colored area) at baseline (**a**) and after interventions (**b**) in one patient for each group.
